# New Mitochondrial Genomic Resources for the Freshwater Fishes of the Sanaga River (Cameroon)

**DOI:** 10.1002/ece3.73663

**Published:** 2026-05-08

**Authors:** Herve Tjomb, Arnold Bitja Nyom, Pierre Caminade, Leah Beche, Khalid Belkhir, Jean‐François Agnese, Nicolas Hubert

**Affiliations:** ^1^ ISEM Univ Montpellier, CNRS, IRD Montpellier France; ^2^ Laboratory of Ecology and Sustainable Development, Faculty of Science University of Ngaoundere Ngaoundere Cameroon; ^3^ Laboratory of Ecosystems and Aquatic Resources; National Higher School of Agronomy, Halieutics and Veterinary Medicine (ENSAHV) Ex‐Institute of Fisheries and Aquatic Sciences University of Douala Douala Cameroon; ^4^ Department of Animal Biology and Physiology University of Yaounde 1, Faculty of Science Yaounde Cameroon; ^5^ Centre D'ingenierie Hydraulique EDF Hydro La Motte Servolex France

**Keywords:** Characiformes, Cichliformes, Cypriniformes, freshwater fishes, mitochondrial genome, phylogenetics, Sanaga River, Siluriformes

## Abstract

The Sanaga River Basin in Cameroon harbors a rich and largely undocumented ichthyofaunal diversity. This study presents the first complete mitochondrial genome dataset for nine freshwater fish species from the basin, spanning four teleost orders: Siluriformes, Characiformes, Cypriniformes, and Cichliformes. A total of 39 specimens were sequenced using Illumina NovaSeq, and mitogenomes were assembled and annotated via a dedicated bioinformatic pipeline. Genome sizes ranged from 16,524 to 16,692 bp, with GC content between 42% and 47%. All mitogenomes exhibited conserved gene structure and order, while the control region (D‐loop) showed notable size variation, consistent with patterns observed in other teleosts. Phylogenetic analyses based on 13 protein‐coding genes and two rRNA genes revealed well‐supported monophyletic clades for each species, confirming taxonomic assignments and validating morphological identifications. This work provides foundational genomic resources for Sanaga basin freshwater fishes and contributes to regional efforts in taxonomy, phylogeography, and conservation.

## Graphical abstract

The Sanaga River Basin in Cameroon hosts a rich but poorly documented freshwater fish diversity. Here, we report the first complete mitochondrial genomes for nine species across four teleost orders, based on 39 specimens sequenced using Illumina NovaSeq. Phylogenetic analyses revealed well‐supported species‐level clades, providing essential genomic references for taxonomy, phylogeography, and conservation in the basin.

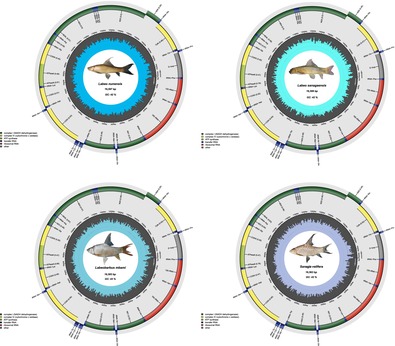

## 1 Introduction

The Sanaga River is the largest river in Cameroon, extending over 900 km (Olivry 1986) and comprising seven dams, three hydroelectric and four storage structures, along its course. Its ichthyofaunal diversity represents an exceptionally rich, yet genetically understudied and increasingly threatened, freshwater diversity. To date, published data on complete mitochondrial genomes of Cameroonian freshwater fishes remain scarce, with only a few studies available from neighboring drainage systems. These include the mitogenome of *Chrysichthys nigrodigitatus* (Lacépède 1803) from coastal rivers near Bamusso (Kim et al. 2018), *Clarias camerunensis* Lönnberg 1895 (De Alwis et al. 2023), and the recent studies on *Enteromius thysi* (Trewavas 1974) and *Coptodon camerunensis* (Lönnberg 1903) from the Nyong Basin (Kundu et al. 2022, 2023).

This study provides the first genomic reference dataset for nine fish species from the Sanaga river, based on the complete characterization of their mitochondrial genomes. These species were selected due to their conservation status, commercial importance, or their relevance for comparison with closely related species which mitochondrial genomes have already been sequenced. Among them, five are endemic to the Sanaga basin: *Sanagia velifera* Holly 1926 classified as Near Threatened by the International Union for the Conservation of Nature (IUCN 2025); *Labeobarbus mbami* (Holly 1927) listed as Endangered; as well as *Labeo sanagaensis* Tshibwabwa 1997; *Labeo nunensis* (Pellegrin 1929) and *Coptodon cameronensis* (Holly 1927), all listed as least‐concern. *Coptodon nyonganus* (Thys van den Audenaerde 1971) was also included to enable comparison with the morphologically similar and closely related species to *C. cameronensis* , originally described from the neighboring Nyong river and present across southern Cameroon, Equatorial Guinea, and Gabon. Other species analyzed include *Alestes macrophthalmus* Günther 1867, *Schilbe mystus* (Linnaeus 1758), and *Brycinus macrolepidotus* (Valenciennes 1850), which have substantial commercial value due to their abundance and importance for local fisheries and food security. Unlike the five taxa restricted to the Sanaga river, the four remaining species exhibit broad continental distributions across multiple ichthyogeographic provinces (Roberts 1975), including the Nilo‐Sudan, Upper and Lower Guinea, and Congo provinces. *Schilbe mystus* in particular has an even wider range, extending to the East Coast, Zambezi, and Quanza provinces. With the objective to improve the availability of mitochondrial genomic resources for this ichthyofauna, we performed Illumina sequencing of total genomic DNA to sequence complete mitochondrial genomes for these nine emblematic fish species from the Sanaga River.

## 2 Material and Methods

### 2.1 Sampling and Morphological Identifications

The specimens analyzed in this study were derived from sampling efforts conducted by the coauthors in the area to assemble a DNA barcode reference library for all the freshwater fishes of the Sanaga river. Specimens were collected from various sites along the Sanaga River and its main tributaries, encompassing the upper, middle and lower portions of the watershed. In total, 39 specimens representing the nine species were sampled between 2023 and 2024 using gill nets and electrofishing. The samples included *Coptodon cameronensis* (*n* = 4), *Coptodon nyonganus* (*n* = 1), *Alestes macrophthalmus* (*n* = 5), *Brycinus macrolepidotus* (*n* = 5), *Labeo sanagaensis* (*n* = 5), *Labeo nunensis* (*n* = 5), *Labeobarbus mbami* (*n* = 4), *Sanagia velifera* (*n* = 5), and *Schilbe mystus* (*n* = 5) (Table S1). *All sampling campaigns were conducted under official permits granted by the Cameroonian Ministry of Scientific Research and Innovation* (No. 166–169/MINRESI/BOO/COO/C10/C13*), the Ministry of Forestry and Wildlife* (No. 2376/PRBS/MINFOF/SETAT/SG/DFAP/SDVEF/SC/ENJ*) for sampling within national parks and* the Ministry of Environment, Nature Protection and Sustainable Development (Prior Informed Consent N°00054/D/MINEPDED/CAN and ABS permit N°00008/MINEPDED/CAN/NP‐ABS/ABS‐FP). Morphological identifications were performed following the ichthyological keys of Stiassny et al. (2007), and were subsequently verified through comparison with a large cytochrome c oxidase subunit I (COI) reference library encompassing a broader taxonomic coverage than the present study (Tjomb et al. *in prep*). This initial COI library was previously compared with NCBI GenBank records corresponding to conspecific or closely related taxa.

### 2.2 DNA Extraction, Sequencing and Genome Assembly

Genomic DNA was extracted using the BioBasic kit, following the manufacturer's recommendations. The quality and concentration of extracted DNA were assessed using NanoDrop spectrophotometry. Sequencing was performed on the Illumina NovaSeq platform, generating paired‐end reads of 150 bp, which were processed using an automated pipeline dedicated to mitochondrial genome assembly and annotation. The initial assembly was performed using MEGAHIT v1.2.9 (Li et al. 2015). Mitochondrial scaffolds were identified using the FindMitoScaf module of MITOZ v3.6 (Meng et al. 2019), enabling the extraction of candidate mitochondrial sequences from the assembled contigs. These mitochondrial scaffolds were subsequently annotated using the AnnotateReads module of MitoZ v3.6 (Meng et al. 2019). We further checked the reliability and mitochondrial origin of the reconstructed sequences using BLASTn v2.14.0 (Altschul et al. 1990). Taxonomic validation of the sequences was conducted by comparisons with the local COI reference library of the freshwater fishes of the Sanaga river (Tjomb et al., in prep). Quality control metrics, including read quality, assembly statistics (e.g., contig length and coverage), and completeness of mitochondrial gene content, were assessed and summarized using MultiQC v1.13 (Ewels et al. 2016). Circular mitogenomes were visualized using Chloroplot (Zheng et al. 2020).

### 2.3 Phylogenetics Reconstructions

Phylogenetic analyses were conducted using concatenated datasets comprising of the 13 protein‐coding genes (PCGs) and two ribosomal RNA genes (12S and 16S) from the complete mitogenomes. The dataset included both the newly assembled sequences from this study and additional mitochondrial genomes publicly available in NCBI GenBank. Phylogenetic trees were reconstructed separately for each of the four orders represented in our dataset, including Characiformes, Cichliformes, Cypriniformes and Siluriformes, using partitioned analyses as implemented in IQ‐TREE v2.2.0 (Minh et al. 2020). The best‐fit substitution model for each partition was automatically selected by ModelFinder (Kalyaanamoorthy et al. 2017) prior to tree inference. Node support was assessed with 5000 ultrafast bootstrap replicates (Hoang et al. 2018), providing robust and computationally efficient estimates of nodes' statistical support. Model parameters were estimated under a shared set of branch lengths while allowing independent evolutionary rates across partitions. The resulting phylogenetic trees were visualized and annotated using FigTree v1.4.4 (Rambaut 2014).

## 3 Results and Discussion

**FIGURE 1:**
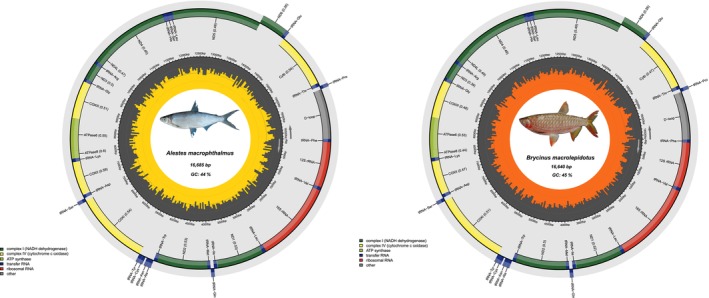
Circular mitochondrial genome maps of *Alestes macrophthalmus* and *Brycinus macrolepidotus* . Each genome includes 13 protein‐coding genes, 22 tRNA genes, 2 rRNA genes, and a control region (D‐loop). Gene orientation is shown by arrows, with colors indicating functional categories.

**FIGURE 2:**
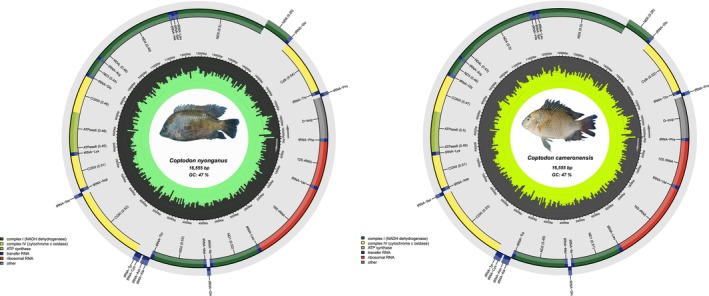
Circular mitochondrial genome maps of *Coptodon nyonganus* and *Coptodon cameronensis*. Each genome includes 13 protein‐coding genes, 22 tRNA genes, 2 rRNA genes, and a control region (D‐loop). Arrows indicate gene orientation; colors denote functional categories.

**FIGURE 3:**
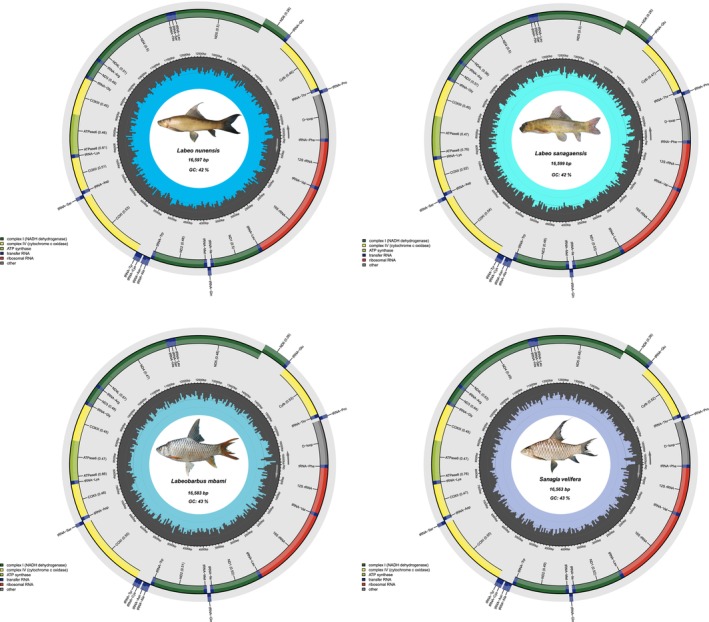
Circular mitochondrial genome maps of *Labeo nunensis* , *Labeo sanagaensis* , *Labeobarbus mbami*, and *Sanagia velifera* . Each genome includes 13 protein‐coding genes, 22 tRNA genes, 2 rRNA genes, and a control region (D‐loop). Gene orientation is shown by arrows, with colors indicating functional categories.

**FIGURE 4:**
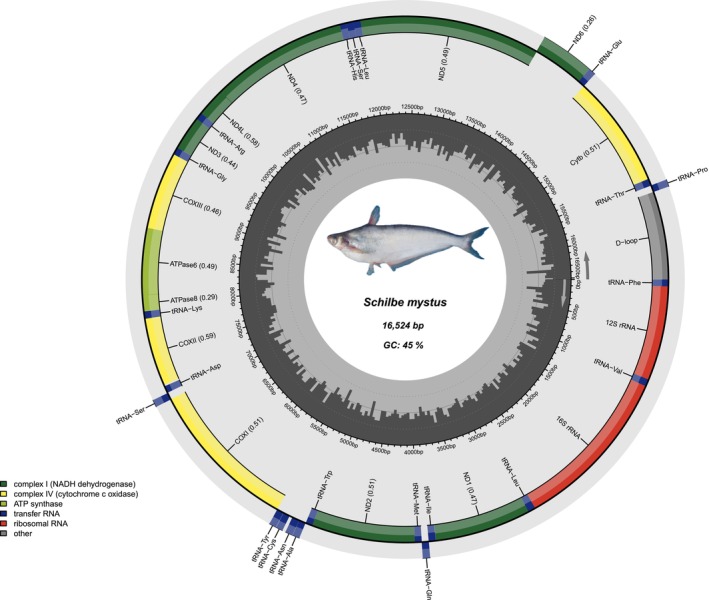
Circular mitochondrial genome map of *Schilbe mystus* . The genome includes 13 protein‐coding genes, 22 tRNA genes, 2 rRNA genes, and a control region (D‐loop). Arrows indicate gene orientation; colors denote functional categories.

We generated complete mitochondrial genomes from 39 specimens representing nine freshwater fish species from the Sanaga river, providing new genomic resources for the West‐Central African ichthyofauna. The assembled mitogenomes ranged in length from 16,524 to 16,692 bp, with a GC content between 42% and 47% (Table S1), values consistent with those previously reported for bony‐fish (Haÿ et al. 2025; Kundu et al. 2022, 2023; Yao et al. 2025). Assembled mitogenomes exhibit the typical structure (Sun et al. 2026) and gene content found in bony fishes (Alvarenga et al. 2024), including 13 protein‐coding genes (PCGs), 22 transfer RNAs (tRNAs), 2 ribosomal RNAs (rRNAs), and a single control region (D‐loop) (Table S2). Despite these species belonging to distantly related lineages, gene order and length remain highly conserved across species. Minor variation in total genome length and GC composition, however, reflects lineage‐specific differences. Alestidae ( *Alestes macrophthalmus* and *Brycinus macrolepidotus* ), for instance, possess the longest mitogenomes (up to 16,689 bp; 44%–45% GC) (Figure 1), whereas the mitogenomes of Cichlidae (*Coptodon cameronensis* and *Coptodon nyonganus*) are slightly shorter and exhibit higher GC content (47%) (Figure 2). The mitogenomes of Cyprinidae (*Labeo nunensis, L. sanagaensis, Labeobarbus mbami*, and *Sanagia velifera* ) show moderate variation (16,560–16,692 bp; 42%–43% GC) (Figure 3), and *Schilbe mystus* (Schilbeidae) displays the shortest genome (16,524 bp; 45% GC) (Figure 4). The control region (D‐loop), in contrast, exhibits notable size variability, ranging from 859 bp in *Coptodon nyonganus* to 1040 bp in *Alestes macrophthalmus* . Such variation is well documented in bony fishes and is generally attributed to the accumulation of tandem repeats and insertion–deletion events within this non‐coding region, which evolves rapidly under relaxed selective constraints (Ma et al. 2015; Tan et al. 2025; Zhou et al. 2024).

**FIGURE 5:**
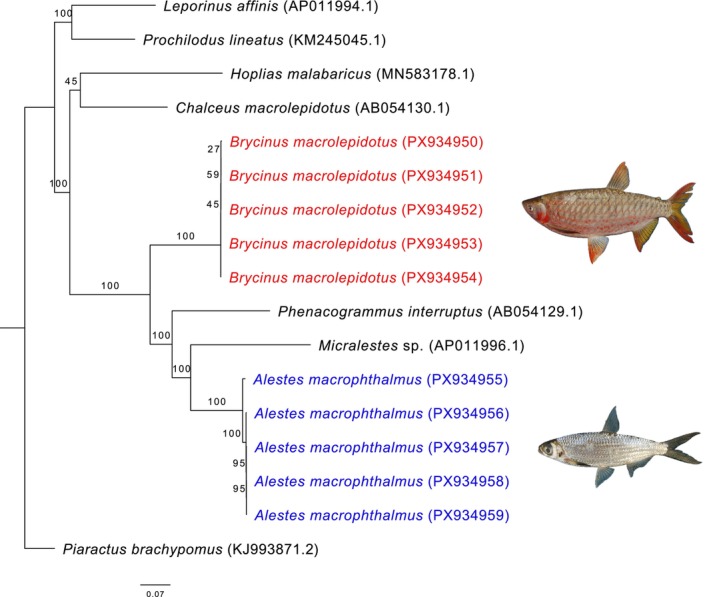
Maximum‐likelihood phylogenetic tree of Characiformes, based on our samples (highlighted) and additional sequences retrieved from GenBank (accession numbers indicated).

**FIGURE 6:**
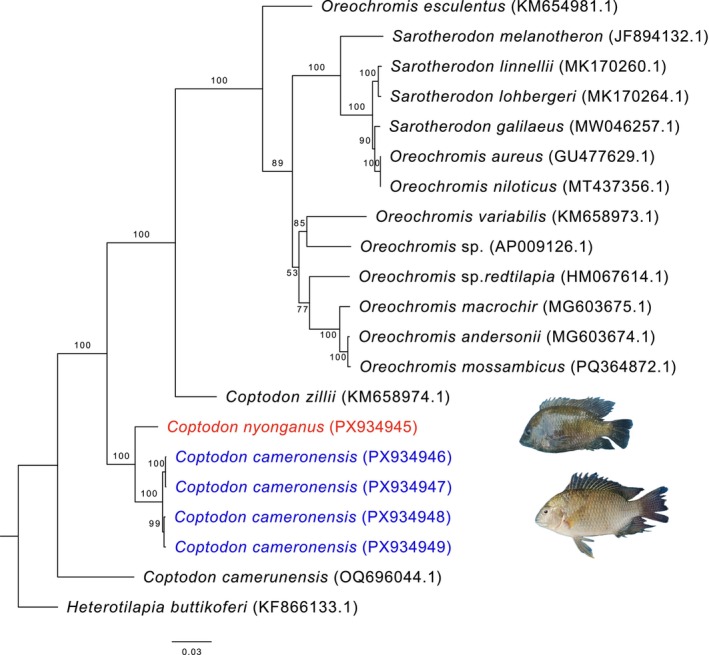
Maximum‐likelihood phylogenetic tree of Cichliformes, based on our samples (highlighted) and additional sequences retrieved from GenBank (accession numbers indicated).

**FIGURE 7:**
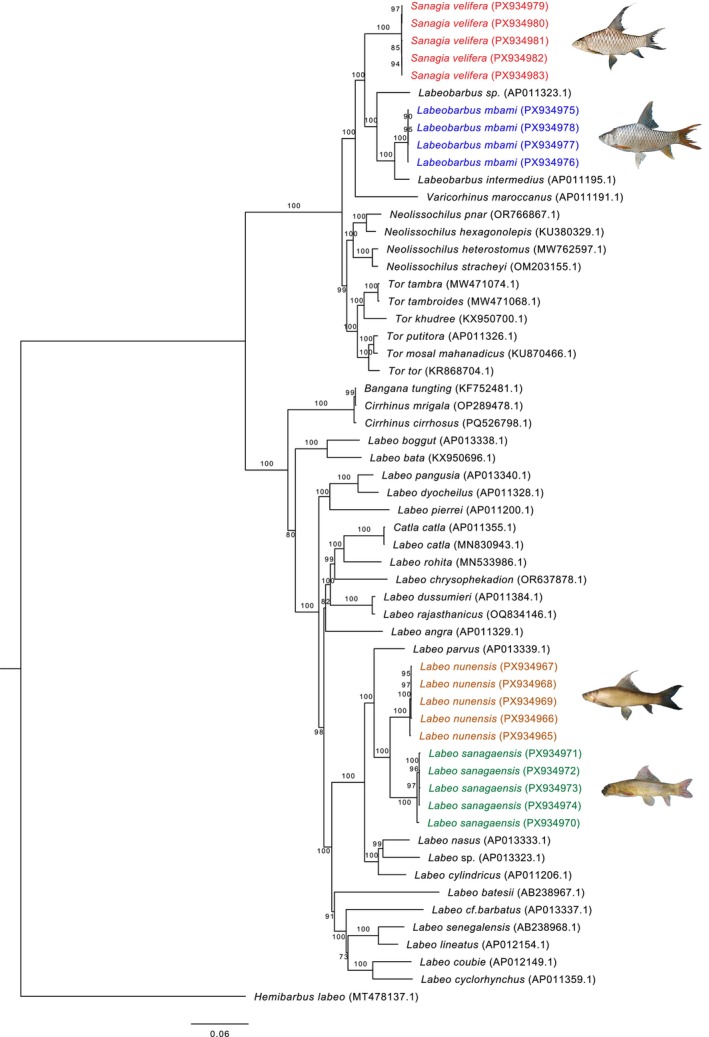
Maximum‐likelihood phylogenetic tree of Cypriniformes, based on our samples (highlighted) and additional sequences retrieved from GenBank (accession numbers indicated).

**FIGURE 8:**
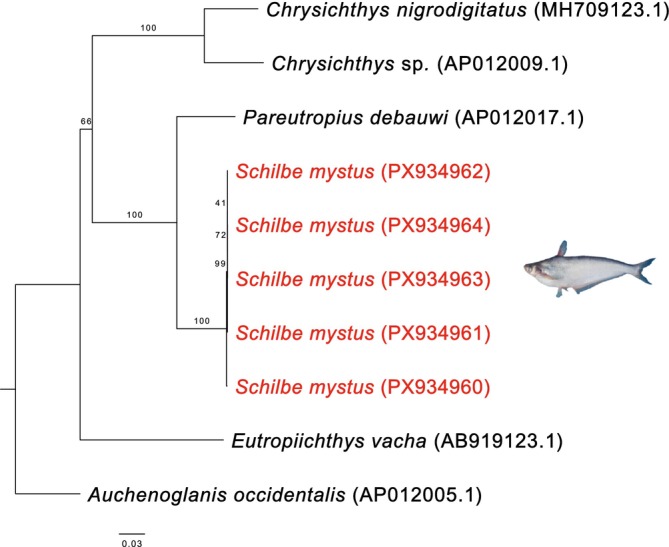
Maximum‐likelihood phylogenetic tree of Siluriformes, based on our samples (highlighted) and additional sequences retrieved from GenBank (accession numbers indicated).

Phylogenetic analyses based on complete mitochondrial genomes were conducted separately for the four teleost orders represented here (Characiformes, Cichliformes, Cypriniformes, and Siluriformes). Across all reconstructions, species consistently formed well‐supported clades, confirming their current taxonomic assignments and validating morphological identifications. Within Characiformes, *Alestes macrophthalmus* and *Brycinus macrolepidotus* were recovered in two distinct, strongly supported clades (Figure 5). In Cichliformes, *Coptodon cameronensis* and *C. nyonganus* each formed independent, well‐supported lineages despite their morphological similarity (Figure 6), highlighting clear genetic divergence within the genus. In Cypriniformes, *Labeo sanagaensis* , *L. nunensis* , *Labeobarbus mbami*, and *Sanagia velifera* clustered into separate, well‐supported lineages (Figure 7), congruent with the morphological identifications. Notably, species of *Labeo* from the Sanaga basin formed a lineage distinct from Asian congeners (*L. angra, L. catla, L. dussumieri, L. rajasthanicus, L. rohita*) and from other African representatives ( *L. cylindricus* , *L. nasus* , *L. parvus*). This pattern underscores the deep evolutionary divergence and long‐term geographic isolation of the Sanaga lineage of *Labeo* within Central Africa. In Siluriformes, all specimens of *Schilbe mystus* clustered tightly within a single, well‐supported lineage (Figure 8), clearly separated from other African representatives of the family Schilbeidae, such as *Pareutropius debauwi* . Overall, the mitochondrial phylogenies exhibit high resolution and strong node support, corroborating the morphological identifications underlying the present study.

## 4 Conclusions

This study represents a significant step forward in the development of mitochondrial resources for freshwater fishes from the Sanaga river in Cameroon. A total of 39 complete mitochondrial genomes were successfully assembled and annotated, representing nine species distributed across four orders: Siluriformes, Characiformes, Cypriniformes, and Cichliformes. For the first time, complete mitochondrial sequences are reported for the genera *Sanagia*, and *Schilbe*, which were previously absent from public genomic repositories. These new genomic resources fill a critical gap in the molecular documentation of African freshwater fishes and provide valuable references for future comparative and evolutionary studies. Overall, this work establishes a foundation for a regional mitochondrial inventory and offers new perspectives for taxonomy, phylogeography, and the conservation of the ichthyofauna of the Sanaga river.

## Author Contributions

**Herve Tjomb:** conceptualization (supporting), data curation (lead), formal analysis (equal), methodology (equal), resources (equal), software (supporting), visualization (lead), writing – original draft (lead), writing – review and editing (equal). **Arnold Bitja Nyom:** conceptualization (equal), funding acquisition (equal), methodology (equal), project administration (lead), resources (equal), writing – review and editing (equal). **Pierre Caminade:** data curation (supporting), formal analysis (equal), resources (supporting), writing – review and editing (equal). **Leah Beche:** funding acquisition (lead), project administration (lead), validation (supporting), writing – review and editing (equal). **Khalid Belkhir:** software (lead), writing – review and editing (supporting). **Jean‐François Agnese:** data curation (supporting), formal analysis (equal), resources (equal), writing – review and editing (equal). **Nicolas Hubert:** conceptualization (lead), data curation (supporting), formal analysis (supporting), methodology (equal), software (supporting), supervision (lead), validation (lead), writing – review and editing (equal).

## Funding

This work was financially supported by the Nachtigal HydroPower Company (NHPC).

## Conflicts of Interest

The authors declare no conflicts of interest.

## Supporting information


**Table S1:** Specimen metadata and mitochondrial genome statistics for nine freshwater fish species from the Sanaga River Basin. The table includes sample ID, taxonomic classification, total mitogenome length (bp), GC content (%), GenBank accession numbers, and GPS coordinates of collection sites.

**Table S2:** Gene structure and orientation of complete mitochondrial genomes for the nine Sanaga Basin fish species. For each of the 37 mitochondrial genes and the D‐loop, the table reports gene length (bp), start (SP) and end (EP) positions, and transcriptional direction (F = forward, *R* = reverse).

## Data Availability

All mitochondrial genome sequences generated in this study have been deposited in the NCBI GenBank database (https://www.ncbi.nlm.nih.gov/genbank/) under accession numbers PX934945–PX934983. Associated metadata and mitogenome statistics are provided in Table S1, while gene structure and orientation details are available in Table S2. Raw sequencing reads have been deposited in the NCBI Sequence Read Archive (SRA) under BioProject PRJNA1453722 (BioSample accession numbers SAMN57272366‐SAMN57272404).
